# CD56–chimeric antigen receptor T-cell therapy for refractory/recurrent rhabdomyosarcoma: A 3.5-year follow-up case report

**DOI:** 10.1097/MD.0000000000017572

**Published:** 2019-10-25

**Authors:** Chiyi Jiang, Wen Zhao, Maoquan Qin, Mei Jin, Lungji Chang, Xiaoli Ma

**Affiliations:** a Beijing Key Laboratory of Pediatric Hematology Oncology, National Discipline of Pediatrics, Ministry of Education, MOE Key Laboratory of Major Diseases in Children, Hematology Oncology Center, Beijing Children's Hospital, Capital Medical University, National Center for Children's Health, Beijing; b Geno-immune Medical Institute, Shenzhen, China; c Department Molecular Genetics and Microbiology, University of Florida, Gainesville, Florida.

**Keywords:** 4SCAR-CD56-CART, refractory recurrent, rhabdomyosarcoma

## Abstract

**Rationale:**

Rhabdomyosarcoma (RMS) is a common soft tissue sarcoma in children with high malignancy. The prognosis of refractory recurrent RMS is extremely poor, and the 5-year survival rate is less than 20%.

**Patient concerns:**

We reported a 2-year-old male patient with RMS who underwent 3 operations and 2 recurrences while being treated with regular multidisciplinary therapy.

**Diagnoses:**

A diagnosis of embryonal rhabdomyosarcoma with primary bladder (IIIa, TNM stage 2, and medium risk group) was made.

**Interventions:**

After repeated recurrence, the patient was treated with chimeric antigen receptor T (CAR-T) cells, which had a safety mechanism and specifically bound the CD56 antigen in the fourth generation.

**Outcomes:**

The process of CAR-T cell transfusion was smooth, and there were no significant cytokine release syndrome manifestations after reinfusion. The patient was in complete remission at last follow-up visit after 3.5 years.

**Conclusion:**

CD56–CAR-T cell therapy is a safe and effective approach and may be an option for children with solid tumors who are nonresponsive to conventional radiotherapy and chemotherapy, or are unsuitable for hematopoietic stem cell transplantation.

## 1 Introduction

Rhabdomyosarcoma (RMS) is the embryonic mesoderm tumor derived from skeletal muscle differentiation. It is the most commonly diagnosed soft tissue sarcoma in pediatric practice (50%–60% of all sarcomas) and is usually found in the head, neck, limbs, urinary system, and the other sites.^[1,2]^ The 2 common pathological types of RMS are embryonal rhabdomyosarcoma (ERMS) and alveolar rhabdomyosarcoma (ARMS).^[3]^ Children with RMS often display different symptoms due to the lack of specific clinical manifestations and the extent to which primary tumor oppresses and invades the surrounding organs and tissues. Therefore, early diagnosis is difficult, and distant metastasis can occur through hematogenic and lymphatic vessels in late stage. Although the survival rate of patients with RMS has increased to greater than 80% due to improvements in diagnosis, imaging, and multidisciplinary treatment approaches, such as surgery, radiotherapy, and combined chemotherapy, the overall prognosis for patients with recurrent and metastatic RMS continues to be poor.^[4]^ Progress in tumor biology and immunology over the years has made cancer immunotherapies dominant in the recent times. Adoptive immune effector cell therapies, particularly chimeric antigen receptor T (CAR-T) cell therapy, have been extensively investigated by top researchers.^[5]^ Here, we report a clinical case of a child with RMS treated with CAR-T cells that have a safety mechanism and bind specifically to CD56 antigen. Periodic evaluations through the 3.5-year follow-up period indicated that the patient continued to be complete remission.

## 2 Case presentation

A 2-year-old male patient presented with a 5-month history of intermittent hematuria and was admitted to our department for the first time on September 4, 2013. He had gross hematuria without obvious inducement 5 months before admission. An ultrasound of the urinary system revealed a 4.8 × 3.1 × 3.9 cm, irregular solid mass in the bladder cavity, abundant blood flow signal in the parenchyma, and tumor tissue invading the posterior urethra. An enhanced computed tomography (CT) of the abdomen revealed a soft tissue density lump shadow in the inferior wall of the bladder. Hence, he was treated with cystourethroscopy, bladder tumor resection, and cystostomy under general anesthesia at the local hospital; no postoperative treatment was administered due to limited treatment conditions. The bladder tumor recurred 3 months later.

Postoperative pathological diagnosis by several hospitals suggested ERMS. The thickened left posterior wall of the bladder observed by postoperative positron emission computed tomography (PET-CT) with radioactive concentration was considered a residual lesion. An enhanced CT of the pelvis suggested that the thickening of the anterior, posterior, and left walls of the bladder were uneven. The thickest spot (about 3.2 cm) was located at the left wall. It showed a nodular soft tissue densification into the lumen. The fat gap between the posterior wall of the bladder and the rectum had disappeared and the adjacent anterior and left walls of the rectum were thickened. The total volume of the bladder lesions was approximately 34.5 cm^3^, and no metastatic lesions were found. According to the clinical grouping of international RMS study group, he was diagnosed IIIa and TNM stage 2 (T2a, N0, M0) before treatment.

Based on BCH-RMS-medium risk group therapy strategy, the tumor was resected a second time after alternative chemotherapeutic treatment with VAC (vincristine 1.5 mg/m^2^, actinomycin D 0.045 mg/kg, cyclophosphamide 2.2 g/m^2^)/VTC (vincristine 0.05 mg/kg, topotecan 1.5 mg/m^2^, cyclophosphamide 750 mg/m^2^) for 7 courses. After operation, VAC/VTC alternate chemotherapy was administered for 4 courses, following which the therapy was upgraded to BCH-RMS-high risk group to administer VDC (vincristine 1.5 mg/m^2^, doxorubicin 30 mg/kg, cyclophosphamide 1.2 g/m^2^)/IE (isocyclophosphamide 1.8 g/m^2^, etoposide 100 mg/m^2^) alternate chemotherapy for 4 courses. Subsequently, local radiotherapy was administered to the abdominal cavity after that (50.4Gy/28f). The local recurrence of cystoma was revealed by imaging after chemotherapy and radiotherapy were stopped for 5 months and 3 months, respectively. Thus, cystectomy was performed again, and VDC/IE chemotherapy was administered for 3 courses after surgery, according to RMS-high risk group. Next, VDC/IE was adopted alternately for 5 courses, followed by 2 courses of CBVP (carboplatin 200 mg/m^2^, etoposide 150 mg/m^2^).

An enhanced CT of the pelvis suggested local thickening of the patient's bladder at the end of the chemotherapy (thickest: 1 cm). Since the patient's tumor was positive for CD56 by pathological immunohistochemistry and to prevent recurrence and remove minimal residual lesion, autologous peripheral blood lymphocytes were collected in the blood transplant ward after explaining the disease to the parents. CD3 cells were isolated from lymphocytes in vitro and then transfected and amplified to prepare the fourth generation CAR-T cells with a safety mechanism and specific binding to CD56 antigen. He received CAR-T cells at the age of 47 months following systemic therapy for 23 months. There was no severe infection before CAR-T cell therapy, complete blood count and organ function were normal, and no obvious tumor. Therefore, preconditioning chemotherapy with CAR-T (cyclophosphamide 250 mg/m^2^/day × 3 days fludarabine 25 mg/m^2^/day × 3 days) was performed 2 weeks after lymphocyte collection. 4SCAR–CD56–CAR-T cells were transfused on September 8, 2015, 2 days after the end of preconditioning chemotherapy. The volume of the retransfused cells was 6  × 10^7^/L, which was equivalent to 3.6  × 10^6^/kg. The patient's vital signs and general status were continuously monitored after retransfusion. Overall, the process of CAR-T cell regurgitation was smooth and there were no cytokine release syndrome (CRS) manifestations such as fever, shivering, rash, hypotension, and pain. After follow-up until January 31, 2019, the patient's tumor status was monitored every month in the first half year after CAR-T cell transfusion and was evaluated regularly every 3 months after 6 months and every 6 months after 1 year. No tumor metastasis or recurrence was found and the thickening of the bladder wall disappeared. He achieved complete remission (CR) according to the Response Evaluation Criteria in Solid Tumors (RECIST). At present, 3.5 years after CAR-T cell treatment, he continues to be in CR and stable.

In addition to monitoring and evaluating the tumor status, the number of CAR-T cells in vivo (with PCR), the levels of the tumor marker lactate dehydrogenase (LDH) and several cytokines and cellular immunity were also regularly tested at early stages after CAR-T cell transfusion. (Table 1)

**Table 1:**
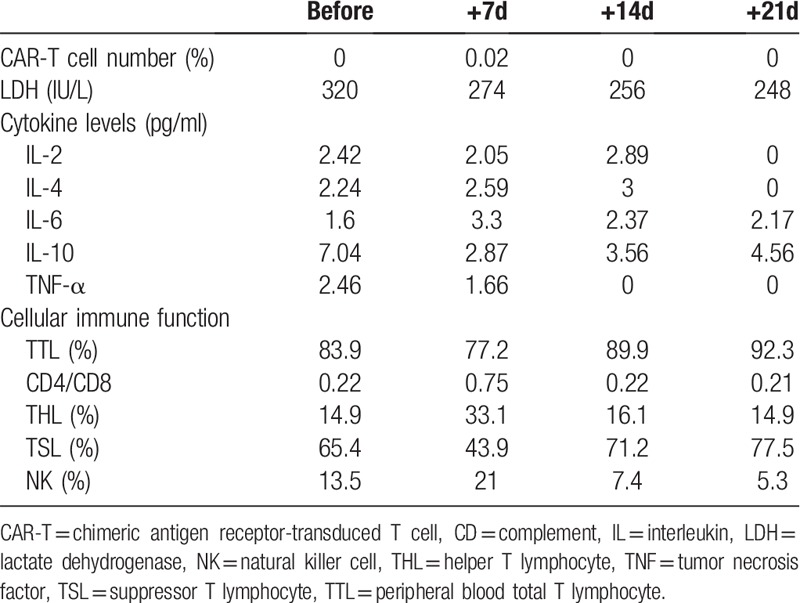
Changes of CAR-T cell number, LDH, cytokine levels and cellular immune function before and after CAR-T.

As shown in the above table, the number of CAR-T cells detected in the patient did not fluctuate significantly since the transfusion, and only 0.02% were observed in the first week after reinfusion. The tested cytokines in the peripheral blood of the patient did not change significantly, consistent with no obvious clinical CRS response. As for cellular immune function, Helper T Lymphocyte decreased about 3 weeks after reinfusion, and Suppressor T Lymphocyte increased.

## 3 Discussion

RMS is a highly malignant soft tissue sarcoma, which can invade the rhabdomytophore tissue and the non-rhabdomytic tissue.^[6]^ The treatment of refractory/recurrent RMS is still a huge challenge, and the long-term prognosis of patients is extremely poor.^[7]^ In recent years, immunotherapy has become a new therapy for tumor.^[8]^

CAR-T therapy immunotherapy technique used to kill tumor cells by genetically modified T-cells.^[9]^ In principle T cells modified by chimeric antigen receptors can kill target cells by specifically recognizing target antigens, independent of the major histocompatibility complex.^[10]^ CD56, a member of the immunoglobulin superfamily is a biomarker of Neural-Cell Adhesion Molecule and NK cells.^[11]^ The positive expression rate of CD56 in skeletal muscle tumors and peripheral neurogenic tumors was significantly higher than that in smooth muscle tumors and other spindle cell tumors.^[12]^

The present case pertains to ERMS in the middle-risk group without distant metastases. However, due to the challenging location of the tumor at the beginning of the disease, it could not be resected completely and recurred shortly radiotherapy and chemotherapy. After 3 repeated operations and 25 courses of chemotherapy, the bladder wall had thickened although there were no discernable residual lesions. Considering the long history, prolonged course of the disease, CD56 positivity, and several recurrences despite combined treatment, the patient was treated by CAR-T cell immunotherapy targeting CD56 to prevent recurrence and to remove minimal residual lesions. The CAR-T cell transfusion process was found to be flawless and safe. The patient was regularly followed up for 3.5 years and his condition had continued to be stable.

In addition, there were 4 other cases of progressed or relapsed bladder/prostate region ERMS in our hospital during the same period (2013–2014). The patients were all boys of ages similar to the patient in the above-mentioned case. In 1/4 case, the patient suffered a local recurrence of the tumor 3 months after chemotherapy was stopped. In the remaining 3 cases, the patients experienced recurrence during their treatment with a median progress time of 6 months. To treat the progressed cancer, they tried the RMS refractory relapse regimen and palliative chemotherapy without CAR-T cell therapy. Unfortunately, the patients did not respond to the treatment and succumbed to the disease.

## 4 Conclusions

Overall, this case report shows that CD56–CAR-T cell therapy is a safe and effective approach for the treatment of refractory/relapsed RMS. Due to limited cases and the low tumor load before CAR-T cell therapy in our case, it is possible that CD56–CAR-T cell therapy may be effective for refractory/relapsed RMS. It may be an option for children with solid tumors who are unresponsive to conventional radiotherapy and chemotherapy or are unsuitable for hematopoietic stem cell transplantation.

## Acknowledgments

We would like to thank the participating patients and their families.

## Author contributions

**Data curation:** Wen Zhao.

**Formal analysis:** Chiyi Jiang.

**Investigation:** Xiaoli Ma.

**Methodology:** Lungji Chang.

**Supervision:** Maoquan Qin.

**Validation:** Mei Jin.

**Writing – original draft:** Chiyi Jiang, Wen Zhao.

**Writing – review & editing:** Chiyi Jiang, Xiaoli Ma.
